# Separate and cumulative effect of risk and protective factors associated with suicidal ideation among Chinese infertile couples: a cross-sectional study

**DOI:** 10.3389/fpsyg.2025.1610027

**Published:** 2025-09-02

**Authors:** Jijie Chen, Jieke Li, Yaqiong Liu, Xiuqing Song, Xiaoyan Yuan, Xuekun Zhang, Xiaoling Deng

**Affiliations:** ^1^The Reproductive Medicine Center, The First Affiliated Hospital of Soochow University, Suzhou, China; ^2^School of Nursing, Suzhou Medical College, Soochow University, Suzhou, China; ^3^Suzhou Key Laboratory of Geriatric Intelligent Nursing and Health Preservation, School of Nursing, Suzhou Medical College of Soochow University, Soochow University, Suzhou, China

**Keywords:** suicidal ideation, infertile couples, infertility-related pressure, anxiety and depression, resilience, marital quality, cumulative effects

## Abstract

**Purpose:**

This study was designed to explore separate and cumulative effects of fertility-related pressure, anxiety, depression, resilience and marital quality on suicidal ideation in Chinese infertile couples.

**Methods:**

A cross-sectional study was conducted among infertile couples at the reproductive medicine center. Suicidal ideation was evaluated using item 9 of PHQ-9. A score of 1 or above indicated the presence of suicidal ideation. The Fertility Problem Inventory, Generalized Anxiety Disorder-7, Patient Health Questionnaire-8, the 10-item Connor-Davidson Resilience Scale and Quality of Marriage Index were used to measure the risk and protective factors. Risk factor index is used to represent the number of risk factors, and protective factor index is used to represent the number of protective factors.

**Results:**

A total of 674 infertile couples participated this study. 65 infertile men and 76 infertile women reported suicidal ideation. Univariate analysis revealed that infertility-related pressure, anxiety, depression, resilience and marital quality were associated with suicidal ideation in both genders. Binary logistic regression revealed positive associations of risk factor index with suicidal ideation in couples (males: OR = 1.966, 95%CI: 1.636–2.363; females: OR = 2.484, 95%CI: 1.992–3.098). The protective factor index was significantly associated with reduced suicidal ideation odds in females (OR = 0.530, 95%CI: 0.316–0.888), but no significant association was found in males (*p* = 0.159).

**Conclusion:**

Our findings indicate that infertility-related pressure, anxiety, depression, resilience and marital quality are associated with suicidal ideation among infertile couples. Specifically, higher risk factor index is linked to increased odds of SI in both genders, with females showing a stronger association. And higher protective factor index is associated with reduced odds of SI in females, though this effect is not significant in males. Therefore, interventions targeting the reduction of infertility-related pressure, anxiety, and depression, coupled with the enhancement of resilience and marital quality, may effectively mitigate the risk of suicidal ideation in this population.

## 1 Introduction

Infertility, defined as the inability to conceive after 12 months of unprotected intercourse, is considered as one of the most pertinent public health concerns worldwide. According to the World Health Organization (WHO), approximately 8–10% of couples, equating to 50–70 million couples are affected by fertility-related problems (Stewart et al., 2019). In China, more than 15 million couples face infertility annually, accounting for 15% of couples of reproductive age (Qiao et al., 2021). This prevalence has been rising due to changes in social structure, lifestyle, and population aging, signaling a global trend (Chow et al., 2016).

Infertility substantially affects both physical and psychological well-being. Beyond hormonal imbalances and health complications from treatments, individuals often endure psychological distress, including shame, frustration, anxiety, and depression (Oexle et al., 2020). Moreover, societal expectations surrounding fertility can further intensify this emotional burden. These expectations include the widespread emphasis on parenthood as a “normal life stage” and the implicit stigma attached to childlessness. This may result in social isolation, financial pressure, and in severe instances, even suicidal ideation (Vander Borght and Wyns, 2018; Reis et al., 2013).

Suicidal ideation (SI), defined as an individual having thoughts of harming themselves without progressing to suicide preparation, is a critical mental health indicator shown to predict actual suicidal behavior (Lotti and Maggi, 2018). Though SI does not always lead to suicide, it reflects profound emotional despair and helplessness. Study has reported that many individuals experience a rapid transition from SI onset to planning or attempting suicide, with this progression often occurring within the first year and potentially resulting in severe injury (Shani et al., 2016). The prevalence of SI varies across different clinical populations. For example, individuals with mental illnesses or chronic conditions, such as cancer, report higher rates of SI than the general population (Fukai et al., 2020; Honrath et al., 2018; Wetherall et al., 2018). Influencing factors of SI, which can be categorized into socio-demographic, disease-related, psychological, and social factors, also vary across group (Cesta et al., 2018; Kolva et al., 2020; Xu et al., 2020). Among these, socio-demographic factors with significant associations, such as income level, residence type, and insurance status, have been identified as key correlates of suicidal ideation in various populations, including those experiencing infertility (Koniares et al., 2022; Wang et al., 2022). Economic strain may exacerbate the financial burden of infertility treatments, while urban–rural disparities in healthcare access can amplify psychological distress, creating a context where socio-demographic disadvantages interact with other risk factors. For infertile couples, unique psychological (e.g., extreme mood swings, self-blame), social (e.g., family pressure), and cultural (e.g., traditional fertility norms) stressors often intersect, exacerbating emotional burden. These overlapping stressors may manifest as intense emotional distress, loss of self-worth, or self-blame—all of which are linked to SI development.

Despite its clinical significance, research on SI in infertile populations remains limited, with most studies conducted in developed countries. In China, the prevalence of SI among infertile couples are particularly under explored. For instance, a review noted that 18.2% of female infertility patients experienced suicidal thoughts following failed artificial insemination (de Castro et al., 2021), while Chen reported 9.4% of infertile patients with SI, attributing childlessness, severe depression, social withdrawal, and self-blame (Shani et al., 2016). However, whether these factors-individually or in combination-sufficiently trigger SI remains unclear.

In addition, previous studies on SI in infertile patients have primarily focused on female infertility in recent years, while male infertility has been comparatively neglected, despite its increasing prevalence and associated psychological burdens. This discrepancy may stem from the fact that women, as the primary bearers of pregnancy and childbirth, are more likely to be the focus of attention and blame. In contrast, men’s emotional experiences, though equally significant, are often overlooked. Understanding gender differences in SI experiences among infertile individuals is critical, as studies across diverse age groups and conditions have consistently shown disparities in both the incidence of SI and factors influencing it (Gmuca et al., 2021; Massarotti et al., 2019; Shani et al., 2016). It has been emphasized that gender differences should be considered when examining SI and its associated factors in infertile patients.

Moreover, previous studies have examined the effects of risk or protective factors on infertile couples using univariate analysis. In reality, however, multiple factors often interact, and their cumulative effects may significantly influence the likelihood of SI. This cumulative effect not only provides novel insights and methodologies for studying factor interaction but also enhance the ecological validity of research (Appleyard et al., 2005). Rutter proposed the cumulative risk model in 1979, advocating for the use of a risk factor index (RFI, defined as the number of risk factors) to quantify the cumulative effect. And Dekovic developed a method to calculate the cumulative effect by integrating RFI and a protective factor index (PFI). Early applications of cumulative effects were documented in studies of adverse childhood experiences influencing children’s cognitive and behavioral outcomes (Anda et al., 2006; Appleyard et al., 2005). In addition, the cumulative risk assumption has since been extended to adult long-term health outcomes and applied to diverse populations, including pregnant women, middle school and college students, and the elderly (Barnes et al., 2022; Salgado García et al., 2020). Research on SI and its influencing factors among infertile couples remains limited, with notable gaps in understanding the interaction of individual and cumulative effects. Despite the psychological complexities associated with infertility, including its potential to lead to severe mental health outcomes, studies exploring these factors remain scarce. Adopting a cumulative effects approach could provide a more comprehensive understanding of how risk and protective factors interact to influence SI. However, few studies have simultaneously modeled the cumulative impact of both risk and protective factors within this population.

In conclusion, given the multifaceted psychological challenges of infertility, including its potential to lead to serious mental health outcomes, there is an urgent need for a more nuanced investigation into both individual and cumulative contributors to SI in infertile couples. This study aims to describe the proportion of SI and explore both individual and cumulative effects of risk and protective factors on SI in Chinese infertile couples. By clarifying these mechanisms, this findings will inform the development of targeted psychological distress screening protocols and evidence-based strategies to mitigate suicide risk in infertile populations.

## 2 Methods

### 2.1 Design and sample

This study is part of a larger study exploring the relationship between mental health and health outcomes in infertile couples. A sample of 820 infertile couples who were planning to receive reproductive treatment were recruited from the First Affiliated Hospital of Soochow University between May 2019 and June 2021. Couples who fulfilled the following criteria were included in the study: (1) older than 20 years as it is the legal marriage age for women and with marriage certification as it is an essential document in order to seek fertility treatment in China, (2) clinically diagnosed with infertility, (3) who can read and write in Chinese fluently, and (4) agreed to participate in this study. Couples with presence or history of mental disorder diagnosed according to the Chinese Classification of Mental Disorder-5, taking antidepressants in the past 6 months, and in which at least one of the parties had biological children were excluded.

**Figure 1:**
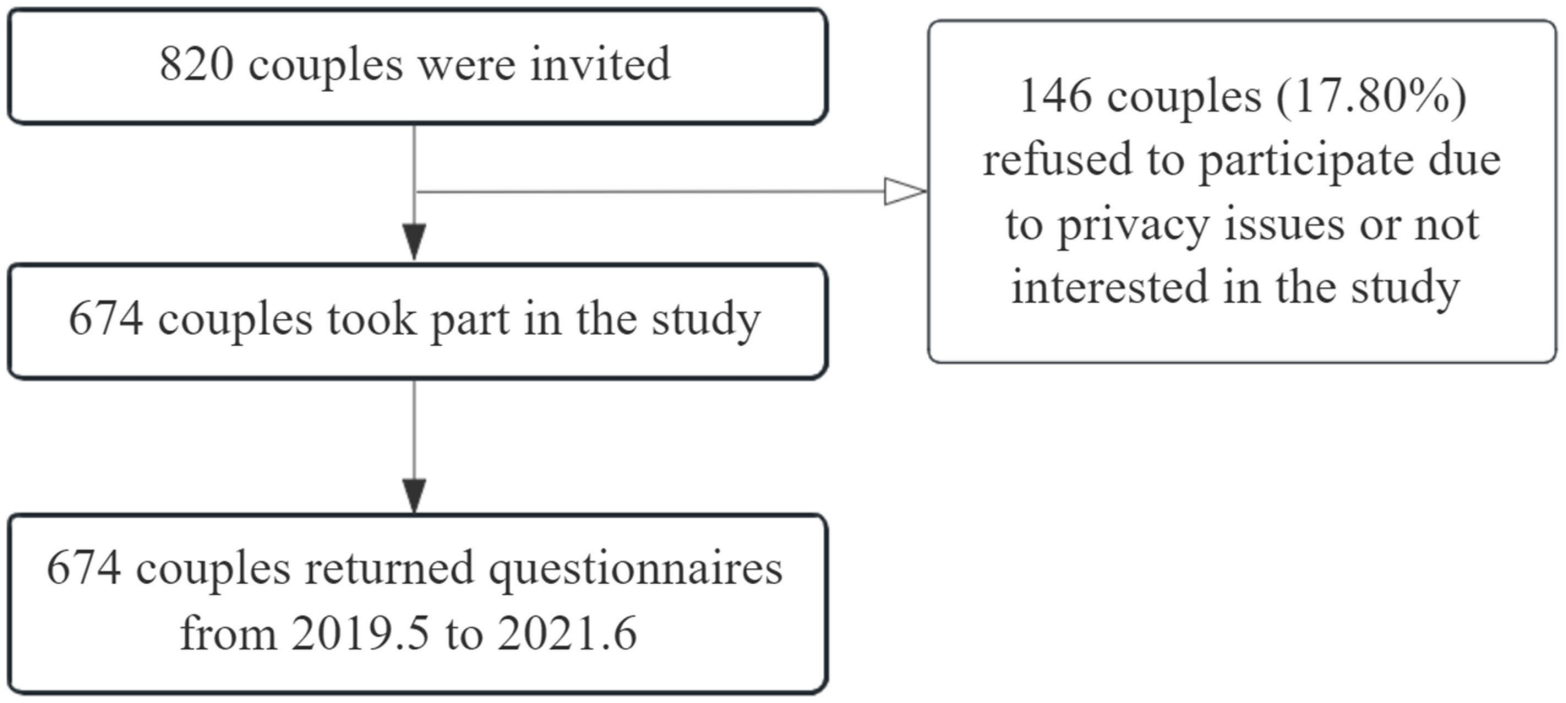
The participant flow diagram.

This study was approved by the Medical Ethics Committee of Soochow University prior to data collection. Trained investigators explained the study’s purpose to participants, who then provided informed consent before enrollment. Questionnaires were self-administered, with couples ensured the opportunity to complete them separately without interference. The participant flow diagram is shown in Figure 1. Approximately 146 couples declined to participate due to privacy concerns or lack of interest. Finally, 674 couples completed the questionnaires. For participants screening positive for SI, researchers would researchers would promptly provide them with access to mental health counseling resources and immediately report relevant information to their attending medical staff.

### 2.2 Measures

#### 2.2.1 Suicidal ideation

Suicidal ideation was measured by Item 9 of Patient Health Questionnaire-9 (PHQ-9) which asks “Over the last 2 weeks, how often have you been bothered by thoughts that you would be better off dead or of hurting yourself in some way?” It was scored on a 4-point scale ranging from 0 (“not at all”) to 3 (“nearly every day”). A score of 1 or higher on PHQ-9 Item 9 indicates the presence of suicidal ideation. PHQ-9 Item 9 has been applied to pregnant women and was validated in the previous studies (Gelaye et al., 2017; Zhong et al., 2016).

#### 2.2.2 Demographic and infertility-related information

A self-designed questionnaire was used to collect the demographic and infertility-related information, including age, educational level, employment, health insurance, type of diagnosis, treatment measure and time since treatment.

#### 2.2.3 Trauma experiences related to infertility

A two-question proxy, which has been used in previous studies to measure trauma, assesses whether respondents’ experience met the Diagnostic and Statistical Manual of Mental Disorders’ criteria for trauma or not (Cordova et al., 2001; Paul et al., 2010). One question is “Did you perceive being diagnosed with and treated for infertility as a threat of death or serious injury or a threat to your physical integrity?” The other question is “Given your experience with infertility, has your response ever involved intense fear or helplessness?” This measure has been documented as reliable and valid with a Cronbach’s *α* of 0.68 (Cordova et al., 2001; Paul et al., 2010). In our study, the Cronbach’s α values were 0.854 among males and 0.727 among females.

#### 2.2.4 Fertility pressure

The Fertility Problem Inventory (FPI) was used to evaluate the fertility-related stress (Newton et al., 1999). It contains 46 items with scores ranging from 1 (“strongly disagree”) to 6 (“strongly agree”). Higher scores indicate greater distress. The Mandarin version of the FPI has demonstrated satisfactory reliability and validity in an infertile Chinese sample, with a Cronbach’s *α* of 0.90 (Peng et al., 2011). The Cronbach’s α vales for FPI was 0.647 for males and 0.633 for females in our study.

#### 2.2.5 Anxiety

The level of anxiety was measured by the Generalized Anxiety Disorder-7 Questionnaire (GAD-7). Each item is scored from0 (“not at all”) to 3 (“nearly every day”), resulting in a total score ranging from 0 to 21, with higher scores indicate greater anxiety severity. A score of 0–4 indicates no anxiety symptoms, while a score of ≥5 indicates the presence of anxiety. The Chinese version of GAD-7 has demonstrated satisfactory reliability and validity in Chinese samples, with a Cronbach’s *α* of 0.84 (Gong et al., 2021). The Cronbach’s *α* of GAD-7 was 0.873 among males and 0.881 among females in our study.

#### 2.2.6 Depression

The first eight items of the Patient Health Questionnaire-9 (PHQ-9) were used to assess depression in infertile couples. Each item is scored from 0 (“not at all”) to 3 (“nearly every day”), and a total score greater than 10 indicates depression. The PHQ-8 has been validated as a reliable measure for assessing depressive mood in adults and pregnant women, with a Cronbach’s *α* of 0.82 (Gelaye et al., 2017; Kroenke et al., 2009). The Cronbach’s *α* of PHQ-8 among males in our study was 0.848 and among females was 0.858.

#### 2.2.7 Resilience

The 10-item Connor-Davidson Resilience Scale (CD-RISC-10) was used to assess resilience in infertile couples. CD-RISC-10 consists of 10 items, and the total score ranges from 0 to 40. Each item ranges from 0 (“not true at all”) to 4 (“true nearly all of the time”), with higher total scores reflecting greater ability to cope with difficulty. This scale has been validated as a reliable and valid tool in Chinese populations, with a Cronbach’s *α* of 0.92 (Meng et al., 2019). The Cronbach’s α for CD-RISC-10 in our study was 0.913 among males and 0.915 among females.

#### 2.2.8 Quality of marriage

The Quality of Marriage Index (QMI) is a 6-item instrument that evaluates relationship satisfaction. The first five items are scored from 1 (“strongly disagree”) to 7 (“strongly agree”), and the last item is scored from 1 (“extremely low”) to 10 (“extremely high”). The total score ranges from 6 to 45, with higher scores indicating better marital quality. It has been confirmed as a valid and reliable tool in China, with a Cronbach’s α of 0.91 (He et al., 2018). The Cronbach’s α of QMI among males in our study was 0.797 and 0.744 among females.

### 2.3 Analytic strategy

Statistical analysis was performed using SPSS version 25.0. Descriptive statistical analysis included means, standard deviation, frequencies, and percentages. Differences in demographic characteristics, infertility-related factors and trauma experiences, infertility-related stress, anxiety, depression, resilience and marital quality scores were compared using t-tests and Chi-square tests. Logistic regression analysis was used to explore the relationship between the separate and cumulative effects of risk and protective factors and SI.

The statistically significant risk and protective factors in univariate analysis were transformed into dichotomous variables according to certain standards, scoring 0 and 1. Variables such as anxiety, depression, and resilience will be dichotomized based on established cutoff values. For numerical variables without well-defined cutoff points, such as fertility pressure, resilience and marital quality would be categorized based on the 75th percentile score, with a score of 1 for those at or above the 75th percentile and a score of 0 for the rest. Building on previous studies on cumulative effects (Barnes et al., 2022; Salgado García et al., 2020), the study adopts the 75th percentile as the cutoff to identify individuals exposed to higher levels of risk or protective factors. This method enhances the specificity of the high-exposure subgroup, ensuring that more intense factors are precisely captured. This approach facilitates a clearer understanding of how the interplay of multiple factors may exert a more significant influence on SI, thus offering deeper insights into the combined effects of risk and protective factors. And then adding all the dichotomous variables scores of risk factors or protective factors to form RFI or PFI. The cumulative effect was analyzed accordingly. Binary multiple logistic regression analysis was employed to include the significant RFI and PFI to determine the causes associated with SI. Two-sided test was used, and the test level *α* was set at 0.05. Analyses were considered as significant at *p* < 0.05.

## 3 Results

### 3.1 Suicidal ideation

**Figure 2:**
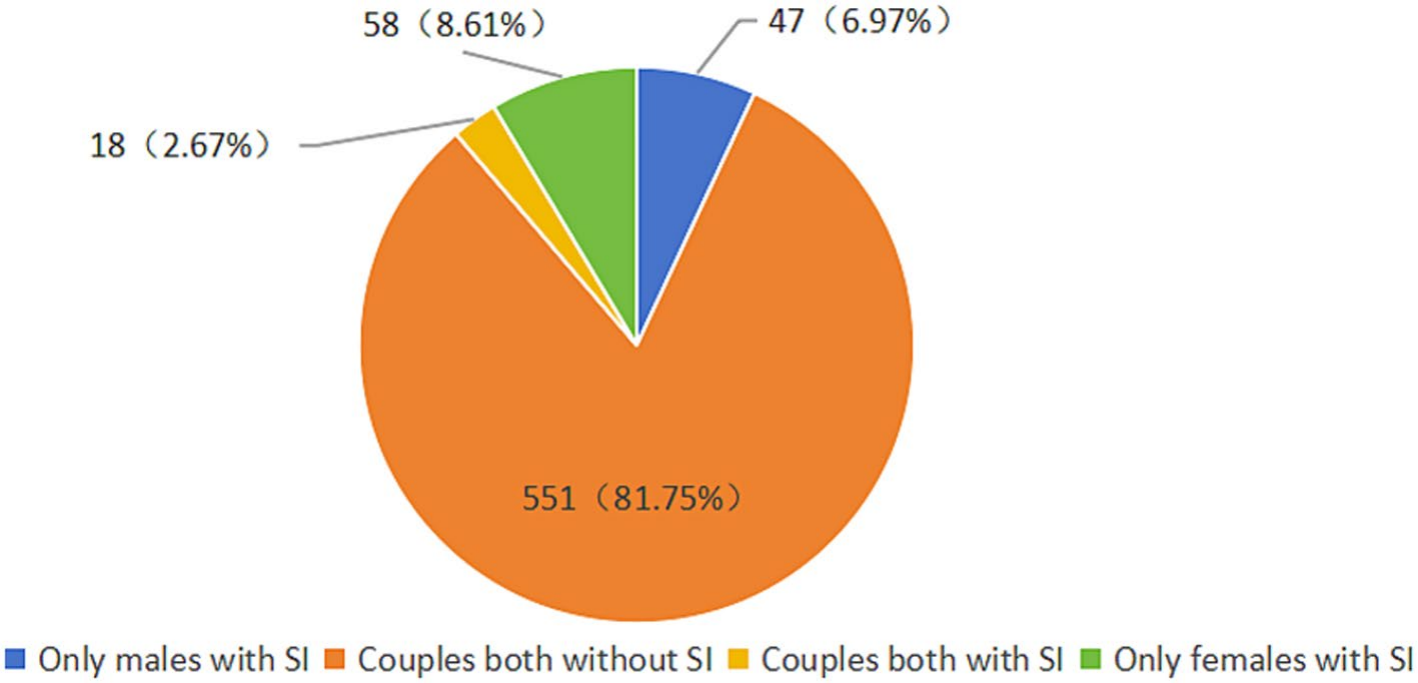
Number of couples with four types of suicidal ideation.

Among 674 infertile couples, 65 (9.6%) infertile men and 76 (11.3%) infertile women screened positive for SI in the past 2 weeks, defined as the PHQ-9 item 9 score 1 or greater. Females SI was higher than that in males, though there was no statistically significant gender difference (χ^2^ = 0.958, *p* = 0.328). Figure 2 illustrates the distribution of couples across the four SI categories in this sample. Specifically, 551 (81.6%) couples had both partners without SI, while 18 (2.7%) couples had both partners with SI.

### 3.2 Separate effect of risk and protective factors

Table 1 presents socio-demographic factors, disease-related factors and self-rated psychological factors. Among males, 122 (18.1%) were aged ≥35 years, 9 (1.3%) self-identified as ethnic minorities, 130 (19.3%) lived in rural areas, 220 (32.6%) had a secondary school education or lower, 190 (28.2%) were low-income (≤5000RMB), and 112 (16.6%) had no medical insurance. Males with SI were more likely to live in rural areas (*p* = 0.028), have lower income (*p* = 0.014), and lacked medical insurance (*p* = 0.026). While in the female group, 85 (12.6%) were aged ≥35 years, 23 (3.4%) self-identified as ethnic minorities, 125 (18.5%) lived in rural areas, 229 (34.0%) had a secondary school education or lower, 78 (11.6%) were unemployed, 400 (59.3%) were low-income, and 130 (19.3%) had no medical insurance. Females with SI were more likely to be low-income (*p* = 0.047). However, there were no statistically significant differences in disease-related factor groupings between the SI group and the non SI group for both males and females. Psychological factors showed that trauma experiences related to infertility (*p* < 0.001), infertility-related pressure (*p* < 0.001), anxiety (*p* < 0.001), depression (*p* < 0.001), resilience (*p* < 0.001), and marital quality (*p* < 0.05) related SI in both males and females.

**Table 1:** Sample characteristics (*N* = 1,348).

Characteristic	Males (*n* = 674)		*p*	Females (*n* = 674)		*p*
	With SI (*n* = 65)	Without SI (*n* = 609)		With SI (*n =* 76)	Without SI (*n* = 598)	
	M ± SD/f (%)	M ± SD/f (%)		M ± SD/f (%)	M ± SD/f (%)	
Socio-demographic factors						
Age, years						
~29	26 (40.0)	207 (34.0)	0.569	36 (47.4)	245 (41.0)	0.549
30–34	27 (41.5)	292 (47.9)		32 (42.1)	276 (46.2)	
≥35	12 (18.5)	110 (18.1)		8 (10.5)	77 (12.9)	
BMI, kg/m						
<18.5	4 (6.3)	16 (2.6)	0.281	4 (5.3)	43 (7.2)	0.449
18.5–23.9	24 (37.5)	264 (43.4)		56 (73.7)	397 (66.5)	
≥24	36 (56.3)	329 (54.0)		16 (21.1)	157 (26.3)	
Ethnic group						
Han	64 (100.0)	600 (98.5)	1.000	75 (98.7)	574 (96.3)	0.461
Minority	0 (0.0)	9 (1.5)		1 (1.3)	22 (3.7)	
Residential area						
Rural	19 (29.7)	111 (18.3)	0.028*	19 (25.0)	106 (17.8)	0.126
Urban	45 (70.3)	497 (81.7)		57 (75.0)	491 (82.2)	
Education						
Secondary school educated or less	27 (42.2)	193 (31.7)	0.09	31 (40.8)	198 (33.2)	0.19
University educated or higher	37 (57.8)	415 (68.3)		45 (59.2)	398 (66.8)	
Occupational status						
Employed	64	606	NA	62 (82.7)	529 (89.1)	0.104
Unemployed	0	0		13 (17.3)	65 (10.9)	
Monthly income (RMB)						
≤5,000	28 (43.1)	165 (27.1)	0.014*	53 (45.1)	347 (58.0)	0.047*
~8,000	20 (30.8)	169 (27.8)		16 (21.1)	114 (19.1)	
~15,000	12 (18.5)	169 (27.8)		3 (3.9)	81 (13.5)	
>15,000	5 (7.7)	106 (17.4)		4 (5.3)	56 (9.4)	
Medical insurance						
Yes	47 (73.4)	511 (84.3)	0.026*	61 (82.4)	476 (80.3)	0.658
No	17 (26.6)	95 (15.7)		13 (17.6)	117 (19.7)	
Disease-related factors						
Diagnosis of infertility						
Primary infertility	38 (62.3)	391 (67.4)	0.419	53 (72.6)	378 (65.6)	0.234
Secondary infertility	23 (37.7)	189 (32.6)		20 (27.4)	198 (34.4)	
Treatment measure						
Ovulation induction	1 (1.5)	9 (1.5)	0.585	1 (1.3)	11 (1.9)	0.801
Artificial insemination	20 (30.8)	225 (37.2)		25 (32.9)	213 (36.0)	
In vitro fertilization	44 (67.7)	371 (61.3)		50 (65.8)	368 (62.2)	
Time since treatment (year)						
0–1	28 (43.1)	282 (46.3)	0.62	33 (43.4)	279 (46.7)	0.594
≥1	37 (56.9)	327 (53.7)		43 (56.6)	319 (53.3)	
History of abortion						
Yes	NA	NA		20 (26.3)	184 (30.8)	0.426
No				56 (73.7)	414 (69.2)	
Psychological factors						
Trauma						
Yes	35 (53.8)	135 (22.2)	<0.001^*^	53 (69.7)	242 (40.5)	<0.001^*^
No	30 (46.2)	474 (77.8)		23 (30.3)	356 (59.5)	
Fertility pressure	164.37 ± 17.96	154.42 ± 14.38	<0.001^*^	162.97 ± 15.79	153.13 ± 14.54	<0.001^*^
Anxiety	8.02 ± 3.88	4.13 ± 3.34	<0.001^*^	9.14 ± 3.96	4.96 ± 3.50	<0.001^*^
Depression	8.65 ± 3.28	3.87 ± 3.34	<0.001^*^	9.99 ± 4.07	4.96 ± 3.50	<0.001^*^
Resilience	24.66 ± 7.35	30.03 ± 6.04	<0.001^*^	22.28 ± 6.95	26.89 ± 6.76	<0.001^*^
Marital quality	36.03 ± 9.06	40.31 ± 7.04	<0.001^*^	34.78 ± 8.10	38.71 ± 7.64	<0.001^*^

SI, suicidal ideation; M, mean; SD, standard deviation; f, frequency; BMI, Body Mass Index.

Have suicidal ideation group: PHQ-9 item 9 score≥1; No suicidal ideation group: PHQ-9 item 9 score<1.

^*^Significant at *p* value < 0.05 by Chi-square of independence/*T* test.

Statistically significant variables ware included in single-factor logistic regression analysis (Table 2). The results showed that among males, living in rural areas (OR = 1.853, 95%CI: 1.045–3.286), lacking medical insurance (OR = 1.916, 95%CI: 1.057–3.474), having low monthly income (OR = 2.036, 95%CI: 1.208–3.434), trauma experiences related to infertility (OR = 4.096, 95%CI: 2.426–6.916), infertility-related stress (OR = 3.573, 95%CI: 2.117–6.031), anxiety (OR = 7.294, 95%CI: 3.739–14.229) and depression (OR = 7.074, 95%CI: 3.786–13.218) were positively associated with SI (*p* < 0.05). Resilience (OR = 0.405, 95%CI: 0.196–0.837), marital quality (OR = 0.446, 95%CI: 0.245–0.812) were negatively correlated (*p* < 0.05). The results for females were similar to those for males.

**Table 2:** Single-factor logistic regression analysis for risk and protective factors.

Characteristic	Males (*n* = 674)		*p*	Females (*n* = 674)		*p*
	OR	95% CI		OR	95% CI	
Risk factors						
Residential area	1.853	1.045–3.286	0.035^*^			
Medical insurance	1.916	1.057–3.474	0.032^*^			
Monthly income	2.036	1.208–3.434	0.008^*^	1.711	1.045-2.804	0.033^*^
trauma	4.096	2.426-6.916	<0.001^*^	3.390	2.024-5.679	<0.001^*^
Fertility pressure	3.573	2.117–6.031	<0.001^*^	3.565	2.185-5.817	<0.001^*^
Anxiety	7.294	3.739-14.229	<0.001^*^	12.254	4.878-30.778	<0.001^*^
Depression	7.074	3.786-13.218	<0.001^*^	8.963	5.259-15.278	<0.001^*^
Protective factors						
Resilience	0.405	0.196–0.837	0.015^*^	0.285	0.128-0.633	0.002^*^
Marital quality	0.446	0.245–0.812	0.008^*^	0.505	0.260-0.983	0.044^*^

OR, odds ratio; CI, confidence interval.

*Significant at *p* value < 0.05.

### 3.3 Cumulative effect of risk and protective factors

The factors with statistical significance in single-factor logistic regression analysis were reassigned based on cumulative effect. Among these variables, residential area was categorized into urban and rural, medical insurance and trauma experiences related to infertility were group into “yes” and “no,” and other continuous variables were divided by cutoffs or the 75th percentile. For males, the RFI scores ranged from 0 to 7, and the PFI scores ranged from 0 to 2. For females, the RFI scores ranged from 0 to 5, and the PFI scores ranged from 0 to 2.

Logistic regression analysis revealed that in males, RFI was positively associated with SI (OR = 1.966, 95%CI: 1.636–2.363, *p* < 0.001). Specifically, each additional risk factor increase in RFI multiplied the odds of SI by 1.966. In females, RFI also showed a positive association with SI (OR = 2.484, 95%CI: 1.992–3.098, *p* < 0.001), with each additional risk factor elevating the odds of SI to 2.484 times the baseline level.

Additionally, PFI exhibited a negative association with SI in females (OR = 0.530, 95%CI: 0.316–0.888, *p* = 0.016), reducing the relative odds of SI to approximately 53% of the baseline, which reflected a protective effect of PFI against SI in females. Conversely, in males, PFI did not demonstrate a statistically significant association with SI (OR = 0.715, 95%CI: 0.448–1.140, *p* = 0.159). Results were showed in Table 3 Model 1.

**Table 3:** Logistic regression models for cumulative effects.

Model	Males (*n* = 674)		*p*	Females (*n* = 674)		*p*
	OR	95% CI		OR	95% CI	
Model 1						
Step 1						
RFI	2.043	1.709–2.443	<0.001^*^	2.530	2.035-3.145	<0.001^*^
Step 2						
RFI	1.966	1.636–2.363	<0.001^*^	2.484	1.992-3.098	<0.001^*^
PFI	0.715	0.448-1.140	0.159	0.530	0.316–0.888	0.016^*^
Model 2						
Step 1						
Residential area	1.327	0.666–2.647	0.422			
Medical insurance	1.372	0.693–2.719	0.364			
Monthly income	1.668	0.912–3.049	0.097	1.689	0.973–2.932	0.063
Trauma	2.339	1.318–4.150	0.004*	1.330	0.736-2.401	0.345
Pressure	1.815	1.013–3.253	0.045*	2.121	1.226-3.670	0.007*
Anxiety	4.162	2.026-8.552	0.000*	6.262	2.376-16.504	0.000*
Depression	2.636	1.322-5.256	0.006*	4.421	2.491-7.846	0.000*
Step 2						
Residential area	1.297	0.648–2.594	0.463			
Medical insurance	1.352	0.681–2.685	0.389			
Monthly income	1.625	0.885–2.984	0.117	1.723	0.988–3.005	0.055
Trauma	2.241	1.249–4.023	0.007*	1.338	0.739-2.423	0.336
Pressure	1.831	1.013–3.310	0.045*	2.180	1.256-3.784	0.006*
Anxiety	4.015	1.941-8.307	0.000*	5.472	2.058-14.551	0.001*
Depression	2.483	1.237-4.986	0.011*	4.369	2.446-7.804	0.000*
Resilience	0.868	0.387-1.948	0.732	0.390	0.164–0.928	0.033*
Marital quality	0.699	0.364–1.341	0.282	0.803	0.382–1.689	0.564

OR, adjusted odds ratio; CI, confidence interval.

*Significant at *p* value < 0.05.

To verify the robustness of the model-based approach, we further directly incorporated the meaningful variables screened by single-factor screening into the multi-factor binary regression model, examined the independent influence of each variable, and compared the results with those of the cumulative exponential model. Results were showed in Table 3 Model 2.

## 4 Discussion

In a cohort of 674 infertile couples, the proportion of SI was 11.3% (76/674) among females and 9.6% (65/674) among males, both of which were higher than the 8% prevalence observed in cancer patients (Ernst et al., 2020). The high proportion of SI in infertile patients may be related to the social stigma, psychological and social pressure, and factors associated with infertility treatment, such as unsatisfactory treatment effect, painful treatment process, great economic pressure and so on (Reis et al., 2013). When individuals experienced perceived psychological burden and negative emotions exceeding their capacity for self-regulation, these factors can be easily lead to desperation, SI and even suicidal behavior. Furthermore, due to the sensitivity of suicide-related issues within the sociocultural context of China, appropriate and feasible tool for measuring suicidal ideation is necessary. So PHQ-9 Item 9 was used to measure SI, and this choice was solely for the purpose of simple screening rather than professional clinical diagnosis.

Our study identified gender differences in SI, with higher proportion in females than in males. This contrasts with findings from the general Chinese population, where suicide rates are higher among males (male-to-female ratio of 1.56), (Zhang et al., 2022). Similar patterns have also been reported in Japan (Liu et al., 2013). Such discrepancies may stem from differences in study populations. In the general population, males often experience less social support due to greater independence and fewer close relationships, increasing their suicide risk. However, there exists a strong correlation between SI and illness. Infertility, as a significant stressor, impacts couples differently, particularly in cultures like China, where reproductive expectations place disproportionate pressure on females due to traditional gender roles. This cultural context may contribute to the higher incidence of SI among infertile females. Additionally, males generally exhibit greater psychological resilience, which may buffer against infertility-related stress and reduce suicidal thoughts. These findings underscore the importance of targeted mental health screening and timely psychological interventions for infertile couples, with particular attention to females’ needs, to mitigate suicide risk and enhance well-being.

In terms of socio-demographic characteristics, our study found no significant associations between age, body mass index (BMI), education, or occupation and SI, which is consistent with findings from a previous study (Oexle et al., 2020). However, economic factors, such as residence, monthly income, and medical insurance, were significantly associated with SI, particularly among males. This indicates that the high medical cost of infertile treatment, which induces a higher finical burden for infertile couples, act as a key stressor.

Psychological factors were significantly associated with SI in both genders. Key factors linked to elevated SI odds included traumatic experiences related to infertility, infertility-related stress, anxiety, and depression. Infertility-related stress scores were notably higher in the SI group, highlighting the need for timely interventions during clinical treatment. Despite this, research on the association between infertility-related stress and SI remains limited, warranting further investigation. Additionally, anxiety and depression were nearly twice as prevalent among those with SI, with this pattern consistent across genders. Anxiety and depression are known to diminish cognitive flexibility, impairing individuals’ ability to consider alternative solutions and potentially increasing the odds of SI (Park et al., 2021). Women exhibited higher rates of anxiety and depression, aligning with findings by Bacigalupe and Martín (2021). This gender disparity may reflect societal norms that discourage emotional vulnerability in males. These findings emphasize the importance of regular screening for anxiety and depression in infertile patients, with early detection and intervention crucial for reducing SI and improving outcomes.

In our study, resilience and marital quality were associated with reduced SI odds in infertile couples. Both male and female patients without SI exhibited higher resilience scores compared to those with SI, consistent with previous studies on the influence of resilience on SI in other groups (Kong et al., 2018). Jakobsen et al.’s (2020) follow-up study found that resilience was a protective factor against SI and suicidal behavior, meaning higher resilience score were associated with lower odd of SI. This align with the mechanism proposed by Gmuca et al. (2021), which is high resilience can buffer against psychological stress from adverse life events, thereby reducing vulnerability to SI. Moreover, we found that of resilience scores of males were higher than those of females, aligning with findings by Bhamani et al. (2020), potentially due to biological and sociocultural influences on emotional coping. Marital quality was significantly lower in the SI group and was associated with SI odds, with better marital quality linked to reduced SI. This aligns with studies in other patient populations, such as those with fibromyalgia, where poor marital adjustment correlates with higher SI (Calandre et al., 2021). As a form of social support, strong marital relationships may help individuals better manage the emotional impact of infertility, promoting acceptance and reducing psychological distress over time.

Regression analyses of the cumulative risk index and cumulative protective index revealed that the odds of SI were associated with the cumulative effect of these indices, with gender-specific patterns. Overall, the cumulative effect model confirmed that factors influencing SI do not act in isolation but interact interact synergistically, consistent with findings in youth populations (Turner and Colburn, 2022). This supports the utility of composite indices in quantifying cumulative effects, aligning with prior research demonstrating that risk and protective factor indices effectively capture cumulative effects on mental health outcomes (Anda et al., 2010; Chartier et al., 2010). For the RFI, a consistent positive association with SI was observed across genders, specifically, each unit increase in RFI was linked to multiplied odds of SI. This indicates that the cumulative burden of risk factors, such as infertility-related stress, anxiety and depression, elevates SI odds in both males and females, underscoring the universal impact of accumulated risk in infertile populations.

In contrast, PFI exhibited gender-specific associations with SI. In females, each unit increase in PFI was associated with reduced SI odds, reflecting greater sensitivity to cumulative protective factors. However, in males, PFI showed no significant association with SI, indicating that cumulative protective factors did not significantly reduce SI odds in this group. Several potential mechanisms may explain this gender disparity in PFI’s association with SI. Behaviorally, men tend to adopt avoidant coping strategies when facing infertility stress, which may negate the positive impacts of resilience or social support, which was key components of PFI (Oliffe, 2023). Psychologically, male self-concept is often tightly linked to fertility and familial roles, and infertility-related impacts to self-esteem may override the potential benefits of protective factors, as negative self-perceptions weaken their capacity to buffer psychological stress. Culturally, societal expectations of male “self-reliance” and rigid gender roles may discourage men from utilizing protective factors within the PFI, such as marital quality or social support (Addis and Mahalik, 2003). Even with high marital quality, men may resist emotional reliance due to stigma around vulnerability, which limits the buffering effect of these factors. In contrast, females are more likely to engage with protective factors through active coping, such as seeking emotional support, which allows the PFI to exert its influence more effectively. These findings highlight the need to consider gender-specific dynamics when addressing cumulative factors contributing to SI. Interventions should target the reduction of RFI across genders, as accumulated risk affects both males and females. For females, enhancing resilience and marital quality may effectively mitigate SI risk. For males, alternative strategies, such as addressing cultural barriers to support utilization or developing gender-tailored coping skills, may be necessary to enhance the effectiveness of protective factors. Future research should further explore these mechanisms to refine targeted interventions for infertile populations.

For males, socio-demographic characteristics are immutable, and the protective effects of resilience and marital quality on SI are not significant. Therefore, it is necessary to identify new targets for intervention. For example, exploring modifiable psychological processes or community-based support models that align with male help-seeking preferences could be potential directions. Meanwhile, interventions for infertile couples should address gender-specific targets, while reducing shared risk factors remains a priority, strategies for males and females should diverge based on their distinct responses to protective factors. These findings highlight the need to consider gender-specific dynamics when addressing cumulative factors contributing to SI. Interventions should target the reduction of RFI across genders, as accumulated risk affects both males and females. For females, enhancing resilience and marital quality may effectively mitigate SI risk. For males, alternative strategies, such as addressing cultural barriers to support utilization or developing gender-tailored coping skills, may be necessary to enhance the effectiveness of protective factors. Future research should further explore these mechanisms to refine targeted interventions for infertile populations.

Our study has several limitations. First, as a cross-sectional study, it only allows us to identify associations between the variables and SI, without allowing inferences about causal relationships or the direction of these associations. Second, the use of single-center sampling may lead to selection bias, limiting the generalizability of our findings to infertile populations in other regions. Third, SI assessment relied on a single item from the PHQ-9, which is insufficient to capture the complexity of SI and is not suitable for clinical diagnostic purposes. And using items from the same scale (PHQ-9) for both SI and depression may introduce shared-method variance, potentially inflating their association. Additionally, SI was self-reported, and measurement bias may exist. Some participants might have under reported SI due to cultural stigmatization or social desirability bias. Furthermore, we did not conduct psychiatric interviews to validate the presence of SI, which might have further compromised assessment accuracy. Another notable limitation is the absence of physiological indicators of SI, which could provide a more comprehensive understanding of the underlying biological mechanisms associated with SI. The integration of physiological measures, such as neurobiological markers or hormonal assessments, could offer valuable insights into the interaction between psychological and biological factors in the context of infertility. To address these limitations, future research should adopt multi-center, large-scale, longitudinal cohort designs to explore causal relationships, employ more representative sampling strategies, and utilize comprehensive, validated SI assessment tools. Additionally, future studies could incorporate physiological indicators to strengthen the understanding of SI in infertile populations and improve the accuracy of findings.

## 5 Conclusion

Among infertile individuals, 9.6% of males and 11.3% of females had suicidal ideation. Traumatic experiences related to infertility, infertility-related stress, anxiety, and depression were associated with higher odds of SI in this population, whereas resilience and marital quality were linked to lower odds of SI. Furthermore, these factors exhibited a cumulative effect, specifically, the odds of SI increased with a higher burden of risk factors and decreased with a higher level of protective factors. These findings may offer insights for clinical practice. Given the stability of socio-demographic characteristics, greater attention should be directed toward modifiable psychological factors in infertile couples. Future research should explore strategies to mitigate infertility-related stress, anxiety, and depression, enhance resilience and social support, thereby reducing SI prevalence and preventing suicidal behaviors.

## Data Availability

The datasets presented in this article are not readily available because in this study, the original dataset is not available for sharing due to several restrictions. Due to privacy and ethical considerations, the dataset is restricted from being shared. Requests to access the datasets should be directed to XZ, zhangxuekun0115@suda.edu.cn.
