# Supplementary material for: Separate and cumulative effect of risk and protective factors associated with suicidal ideation among Chinese infertile couples: a cross-sectional study
Source: Front Psychol. 2025 Sep 2;16:1610027. doi: 10.3389/fpsyg.2025.1610027 (PMC12439718; doi:10.3389/fpsyg.2025.1610027)
Supplement: Supplementary file 1 [file Table_1.docx]

Supplementary materials

| **Table1 Composition of RFI and PFI** | | | | |
| --- | --- | --- | --- | --- |
| **Characteristic** | **Male** | | **Female** | |
|  | **0** | **1** | **0** | **1** |
| **risk factor index, RFI** | | | | |
| Residential area | Urban | Rural |  |  |
| Medical insurance | Yes | No |  |  |
| Monthly income | ＞5000 | ≤5000 | ＞3000 | ≤3000 |
| Trauma | No | Yes | No | Yes |
| Fertility pressure | ≤164 | ≥165 | ≤162 | ≥163 |
| Anxiety | 0-4 | 5-21 | 0-4 | 5-21 |
| Depression | 0-9 | 10-24 | 0-9 | 10-24 |
| **protective factor index, PFI** | | | | |
| Resilience | ≤33 | ≥34 | ≤30 | ≥31 |
| Marital quality | ≤44 | ≥45 | ≤44 | ≥45 |
